# Dataset of octocoral assemblages in fore reefs in the northwestern region of Cuba

**DOI:** 10.1016/j.dib.2020.105790

**Published:** 2020-06-02

**Authors:** Néstor Rey-Villiers, Alberto Sánchez, Hansel Caballero-Aragón, Patricia González-Díaz

**Affiliations:** a Centro Interdisciplinario de Ciencias Marinas del Instituto Politécnico Nacional. Av. IPN S/N, Col. Playa Palo de Sta. Rita, Apdo. Postal #592, 23096, La Paz, Baja California Sur, México; b Instituto de Ciencias del Mar, Ministerio de Ciencia, Tecnología y Medio Ambiente (CITMA). Lomay 39, Plaza, CP 10600, La Habana, Cuba; c Comisión Nacional para el Conocimiento y Uso de la Biodiversidad. Liga Periférico Insurgentes Sur, No. 4903, Tlalpan, 14010, Ciudad de México, México; d Centro de Investigaciones Marinas, Universidad de La Habana. Calle 16 No. 114, Playa, CP 11300, La Habana, Cuba

**Keywords:** octocoral assemblages, zooxanthellate octocoral richness, composition and density of octocorals, fore reefs, Cuba

## Abstract

This dataset accompanies "Spatio temporal variation in octocoral assemblages along a water quality gradient in the northwestern region of Cuba" [1]. Sampling units were quadrats of 1 m^2^ (each 1 x 1 m), positioned by a random-systematic design at 10 m depth on the rocky-coral substrate. The number of colonies of octocoral species in thirteen fore reefs was counted to determine the composition, richness and abundance (expressed as density) of octocorals for the period from 2008-2015. Density of six species that most influenced spatial variation of octocoral assemblage structure was compared [1]. Species richness of octocorals was determined in each reef and for the 2008-2015 period. The accumulated species richness was calculated based on 100 randomizations without replacement. This dataset can be used in meta-analysis studies on spatial variations of the structure of octocoral assemblages related to anthropogenic activities and climate variability in the Caribbean Sea, as well as, experimental studies on thresholds to specific pollutants.

Specifications Table

**Table:** 

Subject	Ecology
Specific subject area	Marine ecology. Sessile benthic invertebrate in fore reefs. Evaluation of the octocoral assemblages.
Type of data	Tables and graphs.
How data were acquired	Underwater sampling via Scuba diving in quadrats of 1 m2 (each 1 x 1 m) was performed.
Data format	Raw and analyzed.
Parameters for data collection	Data collection occurred at an approximate depth of 10 m in the fore reefs near polluted river basins in the northwestern region of Cuba.
Description of data collection	Samplings were performed at a 10 m depth in thirteen fore reefs between 2008 and 2015. Most of the colonies were identified at the species level in situ using a key for field identification of octocorals [2]. When the colony identity was unknown, a branch fragment was cut and placed in a numbered plastic bag for identification in the laboratory based on the shape and size of the spicules [3]. The number of colonies by octocoral species in quadrats of 1 m2 (each 1 x 1 m) was counted. We obtained the data on composition, density (colonies/m2) and richness of octocorals.
Data source location	Data were gathered in thirteen fore reefs along the northwestern shoreline of Cuba over the span of three provinces (La Habana, Artemisa and Mayabeque). Sites of fore reefs in La Habana province: Bajo de Santa Ana (23° 4′27.05″ N, 82° 31′35.05″ W) 1) Jaimanitas (23° 5′43.47″ N, 82° 29′35.73″ W) 2) Club Habana (23° 5′52.30″ N, 82° 28′35.22″ W) 3) Instituto de Oceanología (23° 5′51.74″ N, 82° 28′16.82″ W) 4) Submarine sewage outfall of 180 street (23° 5′51.53″ N, 82° 28′4.15″ W) 5) 70 street (23° 7′1.01″ N, 82° 26′17.98″ W) 6) 30 street (23° 7′28.33″ N, 82° 25′43.57″ W) 7) La Puntilla (23° 8′3.87″ N, 82° 24′55.58″ W) 8) Parque Antonio Maceo (23° 8′34.40″ N, 82° 22′13.15″ W) 9) Cojímar (23° 10′15.66″ N, 82° 17′47.19″ W) 10) Celimar (23° 10′59.00" N, 82° 13′29.00" W) 11) Site of fore reefs in Artemisa province: 12) Salado Beach (23° 2′34.84″ N, 82° 36′26.95″ W) 13) Site of fore reefs in Mayabeque province: 14) Boca de Calderas (23° 10′58.23″ N, 82° 3′14.24″ W)
Data accessibility	Raw data was deposited in the Mendeley repository as a Microsoft Excel file. DOI: 10.17632/8sd6htfy2x.1 http://dx.doi.org/10.17632/8sd6htfy2x.1
Related research article	N. Rey-Villiers, A. Sánchez, H. Caballero-Aragón, P. González-Díaz, Spatio temporal variation in octocoral assemblages along a water quality gradient in the northwestern region of Cuba, Marine Pollution Bulletin, 153 (2020), https://doi.org/10.1016/j.marpolbul.2020.110981[1]

## Value of the data

• The structure of the octocoral assemblages can be useful as bioindicator of organic pollution.
• This dataset is useful for conservation programs and management of coral reefs, and may benefit research on sessile benthic cnidarians.
• This dataset is useful in meta-analysis studies to determine changes in the structure of octocoral assemblages with respect to anthropogenic contamination and climate variability in the Caribbean Sea.
• This dataset is useful for developing ecotoxicology experiments in mesocosms and laboratory in octocoral species to specific pollutants.
• Octocorals have secondary metabolites with antimicrobial properties that could benefit society in the middle and/or long term.

## 1 Data Description

**Fig. 1:**
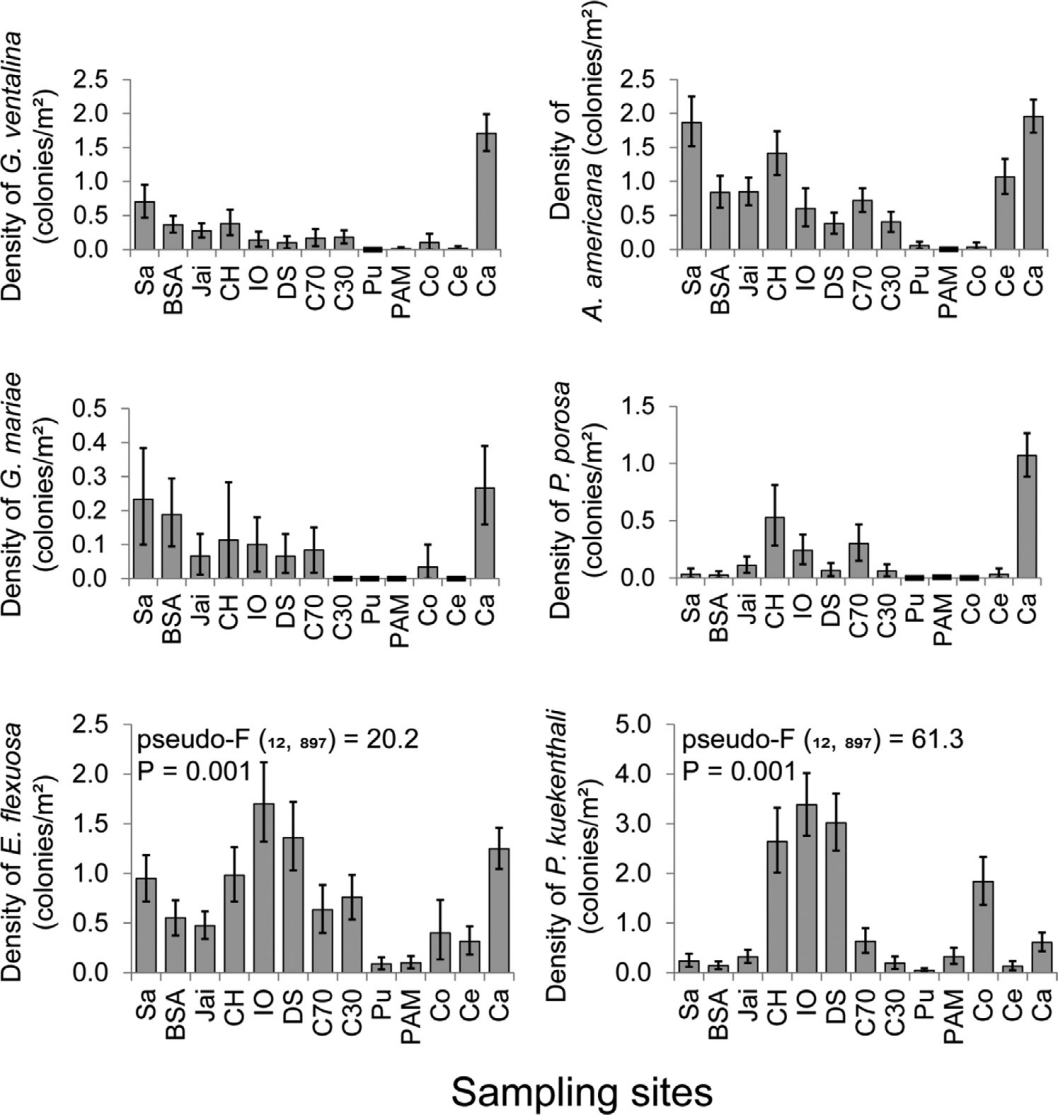
Mean density (colonies/m2) of six octocoral species at the sampling sites from 2008 to 2015. The first four species were not observed at all sites. Overlapping 95% CIs were used as the criterion for nonsignificant differences. The codes for the sampled sites are presented in Table 1.

The data in Table 1 show individual yearly results for the composition, abundance of octocoral species, and total number of colonies in each fore reef in the northwestern region of Cuba. Abundance data consist of mean density (colonies/m^2^) of each octocoral species with respect to the sampled area. Statistical comparisons of mean density (95% confidence intervals, 95% CIs) of the six species that most influenced spatial variation in the multivariate structure of octocoral assemblages can be seen in Fig. 1. Table 2 shows aggregate data over the 2008-2015 period for species richness and sampled area on each fore reef and in the northwestern region of Cuba, which serves as a measure of inventory quality by site.

**Table 1:** Composition and density (colonies/m2) of octocorals in the northwestern region of Cuba (NWRC) from 2008 to 2015. The letters between the brackets are the species codes. PAM = Antonio Maceo Park, Pu = La Puntilla, Co = Cojímar, DS = submarine sewage outfall of 180 Street, IO = Institute of Oceanology, CH = Havana Club, C70 = 70 Street, C30 = 30 Street, Jai = Jaimanitas, Ca = Boca de Calderas, BSA = Bajo de Santa Ana, Sa = Salado Beach and Ce = Celimar.

Species	PAM			Pu			Co	DS			
	2008	2012	2015	2010	2014	2015	2014	2008	2010		

*Briareum asbestinum* (*Basb*)	0.00	0.00	0.00	0.00	0.00	0.00	0.13	0.06	0.73		
*Eunicea calyculata* form *coronata* (*Ecaco*)	0.00	0.00	0.03	0.03	0.10	0.07	0.10	0.48	0.47		
*Eunicea calyculata* form *typica* (*Ecaty*)	0.07	0.10	0.00	0.00	0.20	0.00	0.13	0.23	1.57		
*Eunicea clavigera* (*Ecla*)	0.00	0.00	0.00	0.00	0.00	0.00	0.00	0.65	0.93		
*Eunicea flexuosa* (*Efle*)	0.07	0.07	0.17	0.00	0.20	0.07	0.40	1.42	1.30		
*Eunicea fusca* (*Efus*)	0.00	0.00	0.00	0.00	0.00	0.00	0.00	0.42	0.00		
*Eunicea laciniata* (*Elac*)	0.00	0.00	0.00	0.00	0.00	0.00	0.07	0.00	0.00		
*Eunicea mammosa* (*Emam*)	0.03	0.00	0.00	0.20	0.30	0.40	0.40	0.35	0.60		
*Eunicea tourneforti* (*Etou*)	0.13	0.00	0.00	0.00	0.00	0.00	0.00	0.19	0.07		
*Gorgonia flabellum* (*Gfla*)	0.00	0.00	0.00	0.00	0.00	0.00	0.07	0.00	0.00		
*Gorgonia mariae* (*Gmar*)	0.00	0.00	0.00	0.00	0.00	0.00	0.03	0.06	0.07		
*Gorgonia ventalina* (*Gven*)	0.00	0.03	0.00	0.00	0.00	0.00	0.10	0.03	0.17		
*Muricea elongata* (*Melo*)	0.00	0.00	0.00	0.00	0.00	0.00	0.00	0.10	0.00		
*Muricea laxa* (*Mlax*)	0.00	0.00	0.00	0.00	0.00	0.00	0.00	0.00	0.03		
*Muricea muricata* (*Mmur*)	0.00	0.00	0.00	0.00	0.03	0.13	0.00	0.00	0.13		
*Muricea pinnata* (*Mpin*)	0.03	0.07	0.00	0.00	0.00	0.00	0.03	0.32	0.20		
*Muriceopsis flavida* (*Mfla*)	0.07	0.00	0.03	0.00	0.07	0.00	0.03	0.13	0.17		
*Plexaurella grisea* (*Pgri*)	0.00	0.00	0.00	0.00	0.00	0.00	0.00	0.03	0.03		
*Plexaura homomalla* (*Phom*)	0.00	0.00	0.00	0.00	0.00	0.00	0.00	0.00	0.03		
*Plexaura kuekenthali* (*Pkue*)	0.03	0.40	0.53	0.00	0.10	0.03	1.83	2.61	3.43		
*Plexaurella dichotoma* (*Pdic*)	0.00	0.00	0.03	0.10	0.03	0.13	0.07	0.03	0.07		
*Plexaurella nutans* (*Pnut*)	0.00	0.00	0.00	0.03	0.03	0.00	0.00	0.00	0.07		
*Pseudoplexaura flagellosa* (*Pfla*)	0.00	0.00	0.00	0.00	0.00	0.00	0.00	0.16	0.23		
*Pseudoplexaura porosa* (*Ppor*)	0.00	0.00	0.00	0.00	0.00	0.00	0.00	0.00	0.13		
*Antillogorgia acerosa* (*Aace*)	0.10	0.00	0.00	0.00	0.03	0.00	0.00	0.29	0.00		
*Antillogorgia americana* (*Aame*)	0.00	0.00	0.00	0.00	0.10	0.07	0.03	0.23	0.53		
*Antillogorgia elisabethae* (*Aeli*)	0.00	0.00	0.00	0.00	0.00	0.00	0.00	0.06	0.00		
*Pterogorgia anceps* (*Panc*)	0.00	0.03	0.00	0.03	0.00	0.00	0.07	0.00	0.00		
*Pterogorgia citrina* (*Pcit*)	0.00	0.27	0.07	0.00	0.00	0.00	0.00	0.13	0.07		
*Pterogorgia guadalupensis* (*Pgua*)	0.07	0.17	0.00	0.00	0.00	0.00	0.07	0.06	0.00		

Sampling unit	30	30	30	30	30	30	30	31	30		
Total number of colonies	18	34	26	12	36	27	107	250	331		

Species	IO		CH		C70		C30		Jai		
	2008	2010	2008	2010	2014	2015	2010	2015	2010	2015	

*Briareum asbestinum* (*Basb*)	0.90	2.17	0.74	0.32	0.00	0.00	0.00	0.00	0.00	0.00	
*Eunicea calyculata* form *coronata* (*Ecaco*)	0.15	0.20	0.21	0.12	0.03	0.10	0.15	0.06	0.20	0.12	
*Eunicea calyculata* form *typica* (*Ecaty*)	0.35	1.10	0.47	0.38	0.47	0.23	0.06	0.32	0.27	0.36	
*Eunicea clavigera* (*Ecla*)	0.80	0.33	2.00	0.38	0.00	0.00	0.00	0.00	0.00	0.00	
*Eunicea flexuosa* (*Efle*)	2.00	1.50	1.21	0.85	1.00	0.27	0.88	0.65	0.71	0.28	
*Eunicea fusca* (*Efus*)	0.45	0.00	0.47	0.00	0.00	0.00	0.00	0.00	0.05	0.00	
*Eunicea laciniata* (*Elac*)	0.00	0.00	0.00	0.00	0.03	0.00	0.00	0.00	0.00	0.00	
*Eunicea mammosa* (*Emam*)	0.40	0.47	0.00	0.12	1.07	1.83	2.73	2.91	0.49	0.14	
*Eunicea succinea* (*Esuc*)	0.05	0.03	0.05	0.00	0.00	0.00	0.06	0.00	0.00	0.00	
*Eunicea tourneforti* (*Etou*)	0.15	0.40	0.58	0.29	0.53	0.20	0.39	0.06	0.37	0.08	
*Gorgonia flabellum* (*Gfla*)	0.05	0.00	0.00	0.06	0.00	0.03	0.09	0.00	0.07	0.00	
*Gorgonia mariae* (*Gmar*)	0.10	0.10	0.26	0.03	0.13	0.03	0.00	0.00	0.12	0.02	
*Gorgonia ventalina* (*Gven*)	0.30	0.03	0.16	0.50	0.13	0.20	0.21	0.15	0.27	0.28	
*Muricea elongata* (*Melo*)	0.00	0.00	0.00	0.00	0.00	0.00	0.00	0.00	0.00	0.00	
*Muricea laxa* (*Mlax*)	0.00	0.00	0.00	0.06	0.00	0.00	0.00	0.00	0.00	0.00	
*Muricea muricata* (*Mmur*)	0.05	0.07	0.11	0.29	0.13	0.13	0.09	0.24	0.10	0.20	
*Muricea pinnata* (*Mpin*)	0.25	0.13	0.11	0.00	0.03	0.00	0.18	0.09	0.24	0.00	
*Muriceopsis flavida* (*Mfla*)	0.05	0.13	0.00	0.09	0.53	0.40	0.42	0.24	0.54	0.28	
*Plexaurella grisea* (*Pgri*)	0.00	0.00	0.05	0.03	0.07	0.03	0.18	0.00	0.00	0.00	
*Plexaura homomalla* (*Phom*)	0.00	0.00	0.11	0.00	0.00	0.17	0.03	0.00	0.00	0.00	
*Plexaura kuekenthali* (*Pkue*)	4.70	2.50	4.79	1.44	0.90	0.37	0.09	0.29	0.39	0.26	
*Plexaurella dichotoma* (*Pdic*)	0.05	0.07	0.05	0.12	0.43	0.47	0.67	0.65	0.07	0.08	
*Plexaurella nutans* (*Pnut*)	0.05	0.03	0.00	0.00	0.13	0.13	0.00	0.00	0.02	0.00	
*Pseudoplexaura crucis* (*Pcru*)	0.00	0.00	0.00	0.00	0.03	0.00	0.00	0.00	0.00	0.00	
*Pseudoplexaura flagellosa* (*Pfla*)	0.45	0.27	0.32	0.29	0.30	0.20	0.18	0.38	0.20	0.24	
*Pseudoplexaura porosa* (*Ppor*)	0.40	0.13	0.63	0.47	0.20	0.40	0.06	0.06	0.12	0.10	
*Antillogorgia acerosa* (*Aace*)	0.10	0.03	0.16	0.06	0.03	0.00	0.03	0.00	0.00	0.00	
*Antillogorgia americana* (*Aame*)	0.95	0.37	1.26	1.50	0.63	0.80	0.21	0.59	1.20	0.56	
*Antillogorgia elisabethae* (*Aeli*)	0.00	0.00	0.00	0.00	0.53	0.00	0.03	0.09	0.10	0.02	
*Antillogorgia rigida* (*Arig*)	0.00	0.00	0.00	0.00	0.00	0.00	0.00	0.00	0.00	0.00	
*Pterogorgia anceps* (*Panc*)	0.00	0.00	0.05	0.00	0.00	0.17	0.06	0.00	0.02	0.00	
*Pterogorgia citrina* (*Pcit*)	0.20	0.00	0.16	0.09	0.00	0.00	0.00	0.00	0.10	0.38	
*Pterogorgia guadalupensis* (*Pgua*)	0.05	0.00	0.05	0.00	0.13	0.00	0.00	0.00	0.00	0.00	

Sampling unit	20	30	19	34	30	30	33	34	41	50	
Total number of colonies	260	302	266	255	225	185	225	230	231	170	

Species	Ca			BSA			Sa		Ce		NWRC
	2010	2011	2014	2015	2008	2010	2010	2015	2010	2014	

*B. asbestinum* (*Basb*)	0.00	0.00	0.00	0.00	0.00	0.02	0.00	0.00	0.00	0.00	0.15
*E. caribaeorum* (*Ecar*)	0.04	0.00	0.00	0.00	0.00	0.00	0.00	0.00	0.00	0.00	0.001
*E. calyculata* form *coronata* (*Ecaco*)	0.44	0.33	0.23	0.12	0.06	0.06	0.13	0.20	0.13	0.37	0.16
*E. calyculata* form *typica* (*Ecaty*)	0.59	0.47	0.90	0.77	0.06	0.14	0.30	0.17	1.07	1.07	0.39
*E. clavigera* (*Ecla*)	0.00	0.00	0.00	0.00	0.03	0.00	0.00	0.00	0.00	0.07	0.14
*E. flexuosa* (*Efle*)	1.52	1.43	0.77	1.31	0.72	0.43	1.20	0.70	0.43	0.20	0.71
*E. fusca* (*Efus*)	0.11	0.00	0.00	0.00	0.14	0.00	0.00	0.00	0.00	0.10	0.05
*E. laciniata* (*Elac*)	0.04	0.07	0.17	0.00	0.00	0.00	0.07	0.07	0.03	0.03	0.02
*E. mammosa* (*Emam*)	0.22	0.33	0.30	0.35	0.14	0.18	0.13	0.07	0.23	0.30	0.51
*E. pallida* (*Epal*)	0.00	0.03	0.00	0.12	0.00	0.00	0.00	0.00	0.00	0.00	0.004
*E. succinea* (*Esuc*)	0.04	0.00	0.00	0.00	0.00	0.00	0.00	0.00	0.17	0.00	0.01
*E. tourneforti* (*Etou*)	0.22	0.17	0.00	0.27	0.11	0.14	0.20	0.07	0.77	0.07	0.18
*G. flabellum* (*Gfla*)	1.37	0.43	0.23	0.15	0.42	0.29	0.00	0.00	0.00	0.00	0.11
*G. mariae* (*Gmar*)	0.11	0.37	0.23	0.35	0.31	0.10	0.17	0.30	0.00	0.00	0.10
*G. ventalina* (*Gven*)	0.74	1.53	2.10	2.46	0.36	0.37	0.83	0.57	0.03	0.00	0.39
*M. elongata* (*Melo*)	0.00	0.00	0.00	0.00	0.00	0.00	0.00	0.00	0.00	0.10	0.01
*M. laxa* (*Mlax*)	0.04	0.03	0.00	0.00	0.00	0.00	0.00	0.00	0.13	0.00	0.01
*M. muricata* (*Mmur*)	0.26	0.13	0.10	0.15	0.03	0.02	0.07	0.03	0.07	0.00	0.09
*M. pinnata* (*Mpin*)	0.04	0.03	0.03	0.15	0.00	0.06	0.03	0.10	0.40	0.27	0.09
*M. flavida* (*Mfla*)	0.22	0.50	0.30	0.23	0.22	0.22	1.00	1.57	0.17	0.10	0.27
*P. grisea* (*Pgri*)	0.00	0.00	0.00	0.00	0.00	0.02	0.00	0.07	0.00	0.00	0.02
*P. homomalla* (*Phom*)	0.07	0.10	0.10	0.15	0.03	0.02	0.07	0.00	0.00	0.00	0.03
*P. kuekenthali* (*Pkue*)	0.85	0.57	0.20	0.88	0.19	0.10	0.30	0.17	0.20	0.07	0.84
*P. dichotoma* (*Pdic*)	0.04	0.07	0.00	0.04	0.00	0.02	0.00	0.00	0.07	0.13	0.12
*P. nutans* (*Pnut*)	0.04	0.00	0.00	0.00	0.00	0.00	0.00	0.00	0.07	0.07	0.02
*P. crucis* (*Pcru*)	0.00	0.00	0.07	0.04	0.00	0.00	0.00	0.00	0.00	0.00	0.004
*P. flagellosa* (*Pfla*)	0.00	0.43	0.00	0.00	0.00	0.02	0.00	0.07	0.03	0.00	0.13
*P. porosa* (*Ppor*)	0.96	0.90	1.10	1.35	0.03	0.02	0.07	0.00	0.03	0.03	0.22
*A. acerosa* (*Aace*)	0.00	0.03	0.00	0.00	0.03	0.02	0.03	0.00	0.13	0.67	0.06
*A. americana* (*Aame*)	2.19	1.67	1.90	2.12	1.17	0.59	2.10	1.63	1.10	1.03	0.82
*A. elisabethae* (*Aeli*)	0.04	0.00	0.00	0.00	3.00	0.94	0.57	0.97	0.00	0.00	0.25
*A. rigida* (*Arig*)	0.00	0.00	0.00	0.00	0.00	0.00	0.00	0.00	0.03	0.00	0.001
*P. anceps* (*Panc*)	0.00	0.00	0.00	0.04	0.00	0.00	0.03	0.00	0.03	0.30	0.03
*P. citrina* (*Pcit*)	0.00	0.00	0.00	0.00	0.06	0.02	0.00	0.03	0.00	0.00	0.06
*P. guadalupensis* (*Pgua*)	0.00	0.00	0.00	0.00	0.00	0.02	0.07	0.00	0.13	0.10	0.03
Sampling unit	27	30	30	26	36	49	30	30	30	30	910
Total number of colonies	275	289	262	287	255	188	221	203	164	152	5486

**Table 2:** Evaluation of the inventory quality by site over the 2008-2015 period and in the northwestern region of Cuba (NWRC). The codes for the sampled sites are presented in Table 1.

Site/Period	Species richness	Number of quadrats of 1 m^2^	Slope at the curve end	Registered fauna (%)
Sa 2008-2015	22	60	0.04	93
BSA 2008-2015	25	85	0.04	93
Jai 2008-2015	21	91	0.01	94
CH 2008-2015	27	53	0.05	89
IO 2008-2015	24	50	0.04	94
DS 2008-2015	27	61	0.04	90
C70 2008-2015	25	60	0.03	93
C30 2008-2015	21	67	0.03	91
Pu 2008-2015	12	90	0.02	90
PAM 2008-2015	14	90	0.04	70
Co 2014	16	30	0.14	76
Ce 2008-2015	25	60	0.05	89
Ca 2008-2015	28	113	0.02	94
NWRC 2008-2015	35	910	0.001	94

Raw data was deposited in the Mendeley repository, where they are presented as a Microsoft Excel file composed of 29 sheets (DOI: 10.17632/8sd6htfy2x.1 http://dx.doi.org/10.17632/8sd6htfy2x.1). The name of each sheet is the acronym of the site and year of sampling, and the raw data consist of number of colonies of octocoral species in each quadrat of 1 m^2^ (each 1 x 1 m).

## 2 Experimental Design, Materials, and Methods

### 2.1 Experimental design and methods

Thirteen fore reefs near different river basins were selected along the northwestern coast of Cuba. Four reefs were close to polluted river basins on the Havana coastline, and include Co (near the Cojímar River mouth), PAM (near the entrance channel of Havana Bay), Pu (near the Almendares River mouth) and DS (near the Quibú River mouth). Two reefs were far from these basins and the urban and industrial development of Havana city, and include Boca de Calderas (to the east) and Salado Beach (to the west). Remaining reefs were located at an intermediate distance from each of the nearby river basins. Octocorals were sampled at 10 m depth between 2008 and 2015 using a quadrat of 1 m^2^ (each 1 x 1 m) as the sampling unit. Depending on the octocoral abundance at each site, between 19 and 50 frames were used (Table 1). The quadrats of 1 m^2^ were placed in a random-systematic manner on the rocky-coral substrate. In this way, a 100 m long tape measure was extended parallel to the coastline to represent site characteristics as well as possible. Along this tape measure, three random points were selected every 10 segments where the quadrats of 1 m^2^ were positioned. When the quadrats of 1 m^2^ fell on a sand substrate, they were moved to the nearest rocky-coral substrate in front of the researcher during the dive. All octocoral colonies located within each quadrat of 1 m^2^ were counted and identified to species level. Most species were classified *in situ*, and few colonies were identified in the laboratory.

### 2.2 Data analysis

Data were analyzed and processed using descriptive statistics such asx mean density of each species per site and year of sampling (Table 1). Fig. 1 shows the comparisons of mean density of six octocoral species and 95% CIs. In the case of species *Eunicea flexuosa* and *Plexaura kuekenthali*, the density was statistically compared between sites from a one-way analysis of variance based on 999 permutations of the raw data [4]. For this, the PRIMER 6 and PERMANOVA programs were used. Statistical comparisons were made for these species because they were present at all sites. The 95% CIs were calculated with a Monte Carlo test with 10000 replicates and using the percentiles method. Overlapping 95% CIs were considered the criterion of nonsignificant differences among mean pairs. The accumulated species richness by site was estimated with the pooled 1 m^2^ quadrats at each site over the 2008-2015 period. The accumulated species richness was calculated based on 100 randomizations without replacement with the program EstimateS 9.1.0 [5]. The asymptotic tendency of curves was considered representative of species richness. To evaluate the inventory quality, Clench's nonlinear model was selected [6]. In this model, an inventory is considered complete and reliable when there is a slope at the end of a curve that is smaller than 0.1 and more than 70% of fauna are registered [6], as occurred in sampling sites (Table 2).

## Declaration of Competing Interest

The authors declare that they have no known competing financial interests or personal relationships which have, or could be perceived to have, influenced the work reported in this article.
