# Corrigendum to “Dataset of octocoral assemblages in fore reefs in the northwestern region of Cuba” [Data in Brief 31 (2020) 1–7/DIB-D-20-00391]

**DOI:** 10.1016/j.dib.2021.107259

**Published:** 2021-07-08

**Authors:** Néstor Rey-Villiers, Alberto Sánchez, Hansel Caballero-Aragón, Patricia González-Díaz

**Affiliations:** a Centro Interdisciplinario de Ciencias Marinas del Instituto Politécnico Nacional. Av. IPN S/N, Col. Playa Palo de Sta. Rita, Apdo. Postal # 592, 23096, La Paz, Baja California Sur, México; b Instituto de Ciencias del Mar, Ministerio de Ciencias, Tecnología y Medio Ambiente (CITMA). Loma y 39, Plaza, CP 10600, La Habana, Cuba; c Comisión Nacional para el Conocimiento y Uso de la Biodiversidad. Liga Periférico Insurgentes Sur, No. 4903, Tlalpan, 14010, Ciudad de México, México; d Centro de Investigaciones Marinas, Universidad de La Habana. Calle 16 No. 114, Playa, CP 11300, La Habana, Cuba

The authors regret that in Table 1, the site BSA was sampled in the years 2008 and 2010, and it was not sampled in the 2015 year. Data of 2015 in the site BSA belong to site Ca. Therefore, the site Ca has data in the years 2010, 2011, 2014 and 2015 and the site BSA has data in the years 2008, 2010.

The authors would like to apologise for any inconvenience caused.

